# Bridging the gaps in pediatric complex healthcare: the case for home nursing care among children with medical complexity

**DOI:** 10.1186/s12913-024-11235-1

**Published:** 2024-07-15

**Authors:** Caitlin Koob, Sarah F. Griffin, Mackenzie Stuenkel, Kathleen Cartmell, Lior Rennert, Kerry Sease

**Affiliations:** 1 Department of Public Health Sciences, Clemson University, 501 Epsilon Zeta Dr. (Edwards Hall), Clemson, SC 29634 USA; 2 Prisma Health (Bradshaw Institute for Community Child Health and Advocacy), 255 Enterprise Blvd #110, Greenville, SC 29615 USA; 3 Vanderbilt University Medical Center, Nashville, TN 37232 USA; 4 Furman University, Greenville, SC 29613 USA; 5 Executive Director of the Institute for Advancement of Community Health, Greenville, SC 29613 USA

**Keywords:** Children with medical complexity, Healthcare access, Home nursing care, Medicaid, Healthcare expenditures, Pediatric healthcare reform

## Abstract

**Background:**

Children with medical complexity (CMC) comprise < 1% of the pediatric population, but account for nearly one-third of healthcare expenditures. Further, while CMC account for up to 80% of pediatric inpatient hospital costs, only 2% of Medicaid spending is attributed to home healthcare. As a result, the current health system heavily relies on family caregivers to fill existing care gaps. This study aimed to: (1) examine factors associated with hospital admissions among CMC and (2) contextualize the potential for home nursing care to improve outcomes among CMC and their families in South Carolina (SC).

**Methods:**

This mixed-methods study was conducted among CMC, their family caregivers, and physicians in SC. Electronic health records data from a primary care clinic within a large health system (7/1/2022-6/30/2023) was analyzed. Logistic regression examined factors associated with hospitalizations among CMC. In-depth interviews (*N* = 15) were conducted among physicians and caregivers of CMC statewide. Patient-level quantitative data is triangulated with conceptual findings from interviews.

**Results:**

Overall, 39.87% of CMC experienced *≥* 1 hospitalization in the past 12 months. CMC with higher hospitalization risk were dependent on respiratory or neurological/neuromuscular medical devices, *not* non-Hispanic White, and demonstrated higher healthcare utilization. Interview findings contextualized *efforts to reduce hospitalizations*, and suggested adaptations related to *capacity and willingness to provide complex care* for CMC and their families.

**Conclusions:**

Findings may inform multi-level solutions for accessible, high-quality home nursing care among CMC and their families. Providers may learn from caregivers’ insight to emphasize family-centered care practices, acknowledging time and financial constraints while optimizing the quality of medical care provided in the home.

**Supplementary Information:**

The online version contains supplementary material available at 10.1186/s12913-024-11235-1.

## Background

Children with medical complexity (CMC) comprise less than 1-percent of all children and experience the most complex chronic conditions [1, 2]. These children account for up to 33% of pediatric healthcare utilization, largely due to the extensive provider time required for highly specialized care [1–4]. Despite their small proportion of the pediatric population, CMC account for up to 80% of pediatric inpatient hospital costs and acquire average annual healthcare costs from $8,566 to $54,417 nationwide [2]. As a result, CMC’s annual healthcare costs are approximately 14.2 times that of their peers without complex needs [2]. Beyond direct healthcare costs, a majority of caregivers report spending over $1,000 out-of-pocket annually to provide medical care for their CMC, with some families spending up to $5,719 per month [2, 5]. In addition to the well-documented financial burden among families of CMC, 47% of Medicaid spending among CMC is attributed to hospitalizations, compared to 2% on home healthcare [6, 7].

Compounding the financial responsibilities of caring for CMC, these children often experience fragmented healthcare services and informally rely on caregivers to fill systemic care gaps [3, 8, 9]. Caregivers of CMC provide more than 1.5 billion hours of home-based medical care annually, and over half (59%) of caregivers nationwide report *not* receiving necessary home healthcare for their child [6, 10, 11]. As a result, without home nursing care, caregivers of CMC are unable to maintain employment, and families of children and youth with special healthcare needs (CYSHCN) annually forgo an average of $18,000 in earnings nationwide [12]. Over time, reliance on caregivers to fill clinical gaps often inevitably impacts CMC and their families’ socioeconomic situation, increasing the risk of food insecurity, housing instability, and poor parental mental health—all of which are critical social drivers of health (SDOH) [13, 14]. Accessible home nursing care has demonstrated effectiveness in reducing hospitalization costs among CMC, while also demonstrating the potential to decrease out-of-pocket medical expenses and care burden on caregivers of CMC [15, 16]. Further, in South Carolina (SC), family caregivers are not currently provided respite or financial compensation for providing direct medical care for their children, despite an inability to maintain employment due to the lack of accessible home nursing care.

In recent years, there has been more advocating for family-centered care and involving patients and their families in ongoing research to inform changes within the U.S. healthcare system among vulnerable populations [17–19]. More specifically, with the well-documented disparities between CMC and children without complex needs, there is a shift in healthcare economics from spending on illness to an investment-oriented health system that emphasizes prevention and meets the health needs of CMC and their families [17]. In congruence with calls for preventative, inclusive research, studies focused on the implementation and perceived sustainability of evidence-based interventions that promote value-based care may benefit the well-being and quality of life (QoL) of CMC and their families—which is currently lacking [17]. Thus, this study aims to:

1. Examine associations between patient- and community-level factors and hospital admissions among CMC;
2. Understand the potential for home nursing care to address preventable causes of hospital admissions and mediate healthcare expenditures among CMC; and,
3. Identify potential health system adaptations to address the home nursing care gap in SC.

## Methods

**Fig. 1:**
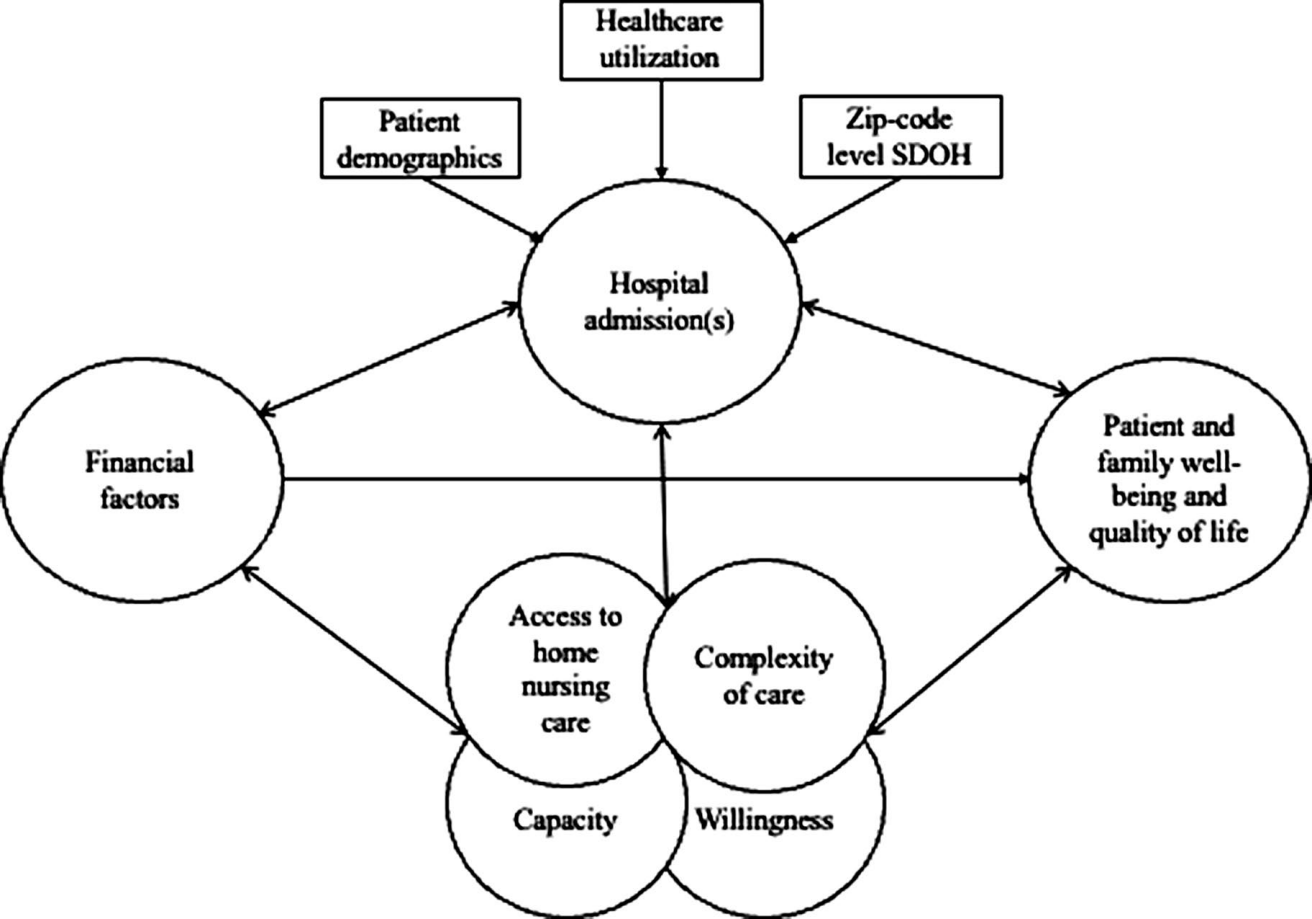
Conceptual model depicting dynamic relationship between patient and family well-being and quality of life, as it relates to accessible home nursing care, and triangulation of patient- and community-level factors

This convergent parallel, mixed-methods study was conducted among CMC, their family caregivers (represented as “caregivers” throughout this study), and primary care physicians in SC [20]. Patient-level quantitative data is contextualized through the triangulation of conceptual findings from interviews with physicians and caregivers of CMC (Fig. 1). This study was approved by the Prisma Health Institutional Review Board (2014123-2).

### Aim 1: sampling, recruitment, and procedures

Quantitative analysis utilized patient-level data from July 2022 through June 2023, sourced from a large healthcare system with a specialized primary care office for CMC. CMC included in this dataset were primarily treated at a specialized primary care office, had *≥* 1 visit during the study period, depended on at least one form of medical technology, and were ages birth through 21 years old. This dataset included 449 unique patients, with 2,943 total primary care visits during the study period.

### Quantitative measures

The outcome of interest was hospital admission (HA) status, measured as whether CMC had at least one HA in the past 12 months. Existing research suggests that hospitalizations significantly influence annual healthcare expenditures among insurance payors and among CMC and their families [2, 5].

Several studies have identified an improved survival rate among CMC over the past 30 years, largely due to advancements in medical technology and improvements in medical care for associated care needs [4, 5]. Therefore, in this study, CMC were grouped for comparison based on dependence on medical device(s) as categorized by body system, using diagnostic (ICD-10) codes (Appendix A). This study applied the following inclusion criteria, depending on which body systems relied on medical devices: neurologic/neuromuscular, cardiovascular, respiratory, renal/urologic, and gastrointestinal. Additional variables consisted of emergency department (ED) utilization in the past 12 months (yes/no) and frequency of primary care visits during the study period. CMC were also characterized by demographic variables, including age (in years, 0 to < 3, 3 to 5, 6 to 11, 12 to 16, 17 to 21), biological sex (male/female), race and/or ethnicity (non-Hispanic White or Otherwise, including non-Hispanic Black, Hispanic, and Other), and Medicaid as primary insurance coverage (yes/no). Age categories were based on existing literature and clinical insight from this research team [21]. Race and/or ethnicity groups were determined based on breakdown within this sample population.

Childhood Opportunity Index 2.0 (COI 2.0), by patient zip code as provided in the electronic health records, was used to examine the effect of community-level factors on CMC’s HA status [22]. COI 2.0 is a validated approach for estimating SDOH, using state-normed data to provide geographic context to healthcare utilization and anticipated longitudinal health outcomes [22, 23]. This study utilized the Social and Economic domain of COI 2.0 and consists of a 3-point range from “low” to “high” opportunity (Appendix B) [22]. COI 2.0 is typically measured on a 5-point scale from “very low” to “very high”; however, we consolidated the scale for this analysis due to sample size and distribution.

### Statistical analysis

Chi-square tests were used to compare differences in HA among independent variables. A logistic regression model was run to identify factors associated with HA among CMC in this sample population. All analyses were conducted using RStudio, version 4.3.0 [24].

### Aim 2: sampling, recruitment, and procedures

Physicians and family caregivers of CMC were recruited statewide to participate in in-depth interviews and provide contextual evidence of home nursing care gaps in SC. Physicians who treat CMC were recruited across the two largest health systems in the state via snowball sampling as physicians identified colleagues and provided contact information. Physicians were evenly recruited across statewide sites (*n* = 6). Following participation, physicians facilitated caregiver recruitment to this study. Physicians invited eligible caregivers to participate during their child’s visit, and then the research team followed up with interested caregivers and scheduled virtual interviews. The interviewer for this study did not have previous relationships with the physicians or caregivers. Eligible participants were (1) primary caregivers of CMC who are *≤* 21 years old, (2) currently reside in SC, (3) whose children receive (or would benefit from) home nursing care, and (4) who speak English. A convenience sample of 9 caregivers was obtained for a total of 15 in-depth interviews. Both physicians and caregivers were included for a well-rounded perspective across home and healthcare environments. Home health nurses could not be reached for study participation due to organizational-level barriers. Interviewees provided verbal informed consent at the time of recruitment and before initiation of the interview.

Based on interviewee preference, physicians and caregivers participated in interviews via Microsoft Teams or telephone, and lasted an average of 30 min and 16 s [25]. Interview guides were developed based on literature review and clinical experience, incorporating questions targeting the priority areas of health equity, well-being of CYSHCN and their families, access and financing of services as detailed in the national agenda for pediatric healthcare reform (Appendix C) [18, 26–28]. Interviews were audio-recorded and transcribed verbatim by an external transcription service.

Transcripts were reviewed, coded, and analyzed, using ATLAS.ti Web, by the research team [29]. Initially, coders employed deductive analysis, developing a preliminary codebook based on the literature, clinical experience, and preliminary data review. Two independent researchers coded four out of 15 transcripts, then compared for discrepancies between application of codes. Coders identified and discussed codes with discrepancies to reach mutual consensus on the appropriate coding scheme. This research team inductively analyzed the qualitative findings throughout the analytic process, adapting the codebook based on a series of data-driven decisions [30]. Major themes and emergent patterns throughout provider and caregiver perceptions were identified and refined to best represent the data and reach final consensus between coders on this research team. Illustrative quotes were selected and presented to support our thematic findings.

## Results

Overall, 39.87% of CMC experienced at least one hospitalization in the past 12 months. Approximately 37% of CMC in this analysis were aged 6 to 11 years and all patients had an average of 6.54 primary care visits (*SD =* 3.92) during the study period. A majority of CMC identified as non-Hispanic White (53.01%), male (55.90%), and were primarily insured by Medicaid (77.73%). Significant differences in hospitalization status were found between those dependent on respiratory (*p* < .001) or neurological/neuromuscular devices (*p* = .019), age (*p* < .001), and ED utilization (*p* < .001), compared to all other CMC (Table 1). Quantitative and qualitative findings were converged to identify patient characteristics at-risk for hospitalization(s) and to consider the potential for high-quality, accessible home nursing care to prevent hospitalizations, mediate healthcare expenditures, and improve health and well-being among CMC and their families.

**Table 1:** Patient characteristics of children with medical complexity who were seen at a specialized primary care office from July 2022 through June 2023 in South Carolina by hospital admission status (*N* = 449)

	Overall (*N* = 449)	No hospitalizations (*n* = 270)	At least 1 hospitalization (*n* = 179)	*p*-value
Reliance on medical technology by body system, *N* (%)				
**Respiratory**				< 0.001
No	305 (67.93)	209 (68.52)	96 (31.48)	
Yes	144 (32.07)	61 (42.36)	83 (57.64)	
**Neurological/Neuromuscular**				0.019
No	397 (88.42)	247 (62.22)	150 (37.78)	
Yes	52 (11.58)	23 (44.23)	29 (55.77)	
**Cardiovascular**				0.217
No	416 (92.65)	254 (61.06)	162 (38.94)	
Yes	33 (7.35)	16 (48.48)	17 (51.52)	
**Renal/Urologic**				0.519
No	424 (94.43)	257 (60.61)	167 (39.39)	
Yes	25 (5.57)	13 (52.00)	12 (48.00)	
**Gastrointestinal**				0.806
No	20 (4.45)	11 (55.00)	9 (45.00)	
Yes	429 (95.55)	259 (60.37)	170 (39.63)	
**Age in years, N (%)**				< 0.001
0 to < 3	102 (22.72)	38 (37.25)	64 (62.75)	
3 to 5	99 (22.05)	55 (55.56)	44 (44.44)	
6 to 11	167 (37.19)	132 (79.04)	35 (20.96)	
12 to 16	55 (12.25)	27 (49.09)	28 (50.91)	
17 to 21	26 (5.79)	18 (69.23)	8 (30.77)	
**Race and/or ethnicity, N(%)**				0.039
Non-Hispanic White	238 (53.01)	154 (64.71)	84 (35.29)	
Non-Hispanic Black	115 (25.61)	68 (59.13)	47 (40.87)	
Hispanic	45 (10.02)	26 (57.78)	19 (42.22)	
Other	51 (11.36)	22 (43.14)	29 (56.86)	
**Sex, N(%)**				0.636
Male	251 (55.90)	148 (58.96)	103 (41.04)	
Female	198 (44.10)	122 (61.62)	76 (38.38)	
**Medicaid as Primary Insurance Coverage, N(%)**				0.705
No	100 (22.27)	58 (58.00)	42 (42.00)	
Yes	349 (77.73)	212 (60.74)	137 (39.26)	
**Emergency Department Utilization, N (%)**				< 0.001
No	223 (49.67)	165 (73.99)	58 (26.01)	
Yes	226 (50.33)	105 (46.46)	121 (53.54)	
**Childhood Opportunity Index 2.0 (Social & Economic Domain, N (%))**				0.335
Very Low/Low	108 (24.05)	68 (62.96)	40 (37.04)	
Moderate	102 (22.72)	55 (53.92)	47 (46.08)	
High/Very High	239 (53.23)	147 (61.51)	92 (38.49)	

Note: *p-values* are calculated via chi-square tests between categorical variables

### Aim 1: predictors of hospitalization status among CMC

#### Healthcare utilization patterns

CMC dependent on respiratory or neurological/neuromuscular device(s), who visited the ED, and attended a primary care visit in the past 12 months were more likely to experience a HA, compared to those who did not utilize the previously mentioned healthcare services (Table 2). CMC dependent on a respiratory (aOR = 1.240, 95% CI: [1.136, 1.354], *p* < .001) or neurological/neuromuscular device (aOR = 1.211, 95% CI: [1.059, 1.386], *p* = .005) were 24.0% and 21.1% more likely to experience a HA, compared to those dependent on renal or urologic device(s). CMC who utilized the ED in the past 12 months were 18.6% more likely to have at least one HA (aOR = 1.186, 95% CI: [1.088, 1.293], *p* < .001), compared to those who did not. According to caregiver and provider insight, ED utilization may be reduced if high-quality home nursing care were available, with the potential to provide ICU-level care in the home environment. With each primary care visit, CMC were approximately 1.8% more likely to experience a HA (aOR = 1.018, 95% CI: [1.007, 1.029], *p* = .002). While this may serve as a severity measure for CMC with an increased need for physician follow-up, the number of primary care visits did not significantly influence the relationship between dependence on medical devices and HA status (*p* > .05).

**Table 2:** Odds ratios to predict hospital admission(s) among children with medical complexity in South Carolina (*N* = 449)

Predictor Variable	Odds Ratio (OR)	95% Confidence Interval	*p*-value
Body system dependent on medical device (Reference: Renal/urologic)			
**Respiratory**	**1.240**	**[1.136, 1.354]**	**< 0.001**
**Neurological/Neuromuscular**	**1.211**	**[1.059, 1.386]**	**0.005**
Cardiovascular	1.129	[0.966, 1.319]	0.128
Gastrointestinal	0.994	[0.808, 1.223]	0.954
Age (Reference: 0 to < 3 years)			
**3 to 5**	**0.828**	**[0.734, 0.934]**	**0.002**
**6 to 11**	**0.728**	**[0.650, 0.815]**	**< 0.001**
12 to 16	0.928	[0.798, 1.078]	0.329
**17 to 21**	**0.764**	**[0.629, 0.929]**	**0.007**
Race and/or ethnicity (Reference: Non-Hispanic White)			
Non-Hispanic Black/Hispanic/Other	1.045	[0.959, 1.138]	0.319
Sex (Reference: Male)	0.967	[0.890, 1.049]	0.416
Medicaid as Primary Insurance Coverage (Reference: No)	0.954	[0.863, 1.055]	0.363
**Primary care visits**	**1.018**	**[1.007, 1.029]**	**0.002**
**ED Utilization**	**1.186**	**[1.088, 1.293]**	**< 0.001**
Childhood Opportunity Index 2.0 (Social & Economic, Reference: High/Very High)			
Very Low/Low	0.995	[0.899, 1.102]	0.929
Moderate	1.039	[0.938, 1.152]	0.462

#### Demographic variables

Compared to CMC aged 0 to < 3 years, CMC who were 3 to 5 years (aOR = 0.828, 95% CI: [0.734, 0.934], *p* = .002), 6 to 11 years (aOR = 0.728, 95% CI: [0.650, 0.815], *p* < .001), and 17 to 21 years (aOR = 0.764, 95% CI: [0.629, 0.929], *p* = .007) were significantly *less* likely to experience HA. Further, caregivers and physicians discussed CMC’s early instability as the healthcare team works with the family to identify and address infants’ complex needs. CMC’s Medicaid insurance coverage (aOR = 0.974, 95% CI: [0.880, 1.078], *p =* .610) or race/ethnicity (aOR = 1.045, 95% CI: [0.959, 1.138], *p* = .319) did not significantly predict HA.

#### Community-level predictors

Nearly 1-in-4 (23.16%) of CMC in this analysis lived in zip codes considered “very low” or “low” in social and economic opportunity, potentially impacting their resource access and household health. However, CMC’s socioeconomic status was not significantly associated with HA (Very Low/Low: aOR = 0.995, 95% CI: [0.899, 1.102], *p* = .929; Moderate: aOR = 1.039, 95% CI: [0.938, 1.152], *p* = .462, compared to High/Very High).

### Aim 2: the case for home nursing care

Three themes and five subthemes were constructed and triangulated with patient-level quantitative data (Fig. 1). All themes contextualized the home nursing care gap and explained its potential to mediate healthcare expenditures, from the perspectives of caregivers and physicians of CMC.

### Theme 1: Participants shared their beliefs about the causes of the shortage of home health nurses and how the shortage affects the care of CMC

Caregivers and physicians of CMC identified *financial factors* and the *capacity of available nurses* as contributing factors to the lack of accessible home nursing care (Table 3). Nurses’ “capacity” was described as a combination of their skill level and/or willingness to provide high-quality medical care to CMC. Compared to inpatient facilities, the lower rate of payment for nurses in the home environment left physicians and caregivers reporting feelings of helplessness due to their inability to change payment models.

**Table 3:** Themes 1 and 2: contextualizing the home nursing care gap and efforts to reduce hospitalizations (*N* = 15)

Theme 1: Participants shared their beliefs about the causes of the shortage of home health nurses and how the shortage affects the care of CMC	
*Themes/Subthemes*	*Illustrative Quotes*
*Financial factors*	“There are plenty of nurses out there, it’s just at the same time do you go work in a home for $22.00 an hour, do you go work in a school for $24.00 an hour, or do you go work in a hospital or as a travel nurse making $125.00 an hour?” – *Caregiver_1* “Financially, it’s not as lucrative for people to go into the private duty nursing realm. They can make more money in as a traveling nurse or as a nurse working on an inpatient unit. And nurses don’t get reimbursed for mileage…I think until Medicaid is paying a competitive rate, I think probably it could always be challenging to staff these areas.” – *Provider_1*
*Capacity of available nurses*	“The medically complex children are seeming to get more medically complex and so it seems like there are more kids leaving NICU and PICU on vents and on trach’s and finding nursing for them is very difficult.” - *Provider_2* “We’ve had people that come into the house that have had background checks that showed not only drug charges but elder abuse, child abuse, everything, and it all stems from the fact that right now in the state of South Carolina, the pay rate for nurses in the home setting is very low.” - *Caregiver_1*
***Theme 2***: Participants share their ongoing efforts to reduce hospitalizations among CMC.	
*Themes/Subthemes*	*Illustrative Quotes*
*Caregiver willingness and capacity to fill the clinical gap*	“If I’m home by myself, I cannot lift both my boys…We’re still waiting on funding to get a home lift that would aid in the process of moving them. And so I cannot be home by myself with the boy without my husband being there.” – *Caregiver_2* “My daughter has a trach…You have to be trained how to suction a trach, what you can do for a trach, what you can’t do for a trach, and some nurses just have no nursing capability to do that. They have no desire to take care of trachs…it doesn’t bother me. But I know seasoned nurses that are like, “Ooh, hold up…Nuh-uh. I’m not suctioning a trach. I’ll do anything…but I will not touch a trach.” – *Caregiver_1* “When we don’t have nursing, or when we didn’t have nursing, our role was pretty much to be like a nurse, not necessarily just a parent. But trying to make sure that we start their feeds on time, planning around their NG tube, changing out their NG tubes when they needed. Especially with trach care, having to do trachea [care] at least once a day. And making sure their oxygen sat. and everything stayed up, because they’ve got the monitors…not just like a typical parent watching your kid play.” – *Caregiver_3* “Oh, all day. I’m going to keep eyes on them.… Her hospital bed is beside my bed, and she actually had a seizure this morning about 5:00, so I can tell by her breathing and her pulse ox going off that she’s having an episode.” – *Caregiver_4* “The ventilator beeps in the middle of the night…I have a set of monitors so I can hear…I don’t sleep so heavy because I listen for the sound of the ventilator running.” – *Caregiver_5* “Home nursing can give mom some respite, because without it she has trouble going to the grocery store and she has difficulty taking care of her other children who might need help with homework or might want to go to dance class or might want to go to baseball games…All of that part of family life is compromised, when there’s no one to even for short periods of time, take care of the complex kid.” – Provider_3 “I think one of the things is a little bit of respite for the parents, especially if they have a nurse that they really bond with and they trust and is reliable and they are comfortable with them taking care of the children. Even if they are still at home, they can go into another room and be with another child or watch a TV show or go take a shower or something that you wouldn’t necessarily be able to do if there wasn’t somebody there taking care of their child. I think that time-wise and mentally, it’s a big help for the parents and physically, to have to do certain things.” – Provider_4
*Inpatient-level care in the home environment*	“It’d keep them out of the hospital. If they are worsening, the nurse is trained to notice that, take them to their primary care specialist and hopefully manage that outpatient versus hospitalization which, PICU, it’s $10,000, $12,000 a day versus just an outpatient visit.” – *Provider_5*
	“Some things that would normally have come to the pediatric floor, we don’t have to admit that child at all if we have enough support at home. That’s particularly true for ventilator kids. If you got a kid with a ventilator, you can almost do an ICU level care at home if you’ve got a home nurse and a good parent.” – *Provider_3* “At times, it can feel like a mini hospital around here when he’s sick, but nursing care has also enabled us to try things at home longer. We don’t try to hospitalize him with every illness. We try to manage them at home, so it needs to be a hospital here at home, and the days when it’s just us and things are going well, like his nurse gave him a spa day recently and put cucumbers on his eyes and his hair was wrapped up in a towel. It’s like the care of the hospital and medical team but the heart of a home all in one place.” – *Caregiver_6*

Beyond financial considerations, nurses who provide home nursing care are responsible for addressing CMC’s complex needs in a non-medical environment. This level of care in a non-medical environment can lead to high-stress levels for nurses, as described by physicians and caregivers. Conversely, while physicians discussed the high quality of home nursing care needed, caregivers repeatedly identified a lack of quality nursing care available for their children and families. Reportedly stemming primarily from the lack of financial compensation, caregivers shared experiences of home nurses’ lack of training, fatigue, burnout, theft, and neglect as examples of their lack of trust in the current system. Caregivers and physicians alike reported systemic barriers to accessible home nursing care for CMC and their families in SC, contributing to the increased susceptibility to experiencing HA among this population.

### Theme 2: participants share their ongoing efforts to reduce hospitalizations among CMC

Due to the complexity of care required, patients with medical devices may be admitted to a pediatric intensive care unit (PICU), rather than a general pediatric HA. Physicians discussed the substantial cost of prolonged PICU stays and the ability to *provide inpatient-level care at home*, which can substantially reduce healthcare expenditures through avoided PICU stays (Table 3).

Further, caregivers were motivated to keep CMC in the home environment as often as possible, citing their desire to avoid hospitalizations and the self-reported *high quality of care provided by caregivers* in the home (Table 3). In many cases, caregivers shared their around-the-clock, physically taxing efforts to fill the home nursing care gap, learning how to provide complex medical care in the home to the best of their abilities. Caregivers reported often being more willing to provide complex care for their children, even with nursing care in place. Among caregivers with home nursing care, some discussed comfort and safety for CMC and their families with trusted nursing support at home, with the potential to improve family well-being and QoL. Still, caregivers shared the difficulties and intermittent medical instability of CMC within their homes, which is partially explained by the complexity of conditions, gap in home nursing care, and the reliance on family caregivers to provide high-quality clinical care with limited support.

### Theme 3: Participants discuss opportunities to improve patient and family well-being and quality of life with accessible, high-quality home nursing care

Per caregivers and physicians, reduced hospitalizations among CMC would improve the well-being and QoL of CMC, their caregivers, and other family members, while also *reducing family financial burden* associated with HA and relevant home-based medical care (Table 4). Some physicians explained that CMC may experience extended HA due to the lack of home nursing care available and that when quality home nursing care is available, hospitalizations may be avoided. Prolonged HA disrupts family functioning for CMC, their caregivers, and siblings, which can significantly affect well-being of the family unit over time and may be exacerbated with repeated admissions. Physicians further described the impact of maintaining care in the home as more valuable than financial considerations alone, benefiting the QoL of patients and their families.

**Table 4:** Theme 3: participants discuss opportunities to improve patient and family well-being and quality of life with accessible, high-quality home nursing care (*N* = 15)

Themes/Subthemes	Illustrative Quotes
*Improve patient and family well-being and quality of life*	“It helps minimize visits to the ER, visits to the doctor if it’s something they can feel manage certain things at home versus if just the parent was there and they’d maybe be a little bit more likely to go to the ER. I think that would be the biggest benefit for the patient. I don’t know if it numbers-wise really makes a difference, outcomes-wise long term. But in the short term, I do feel like it helps.” – *Provider_4* “So it’s a great partnership between the nurses and the doctors because the nurses know the doctors because they come with us to the appointment. And so sometimes if I’m like, I forget what he said. They’re like, oh no, this is what we need to do. And I’m like okay, good.” - *Caregiver_2* “A child may be medically ready for discharge and be kept in the hospital for weeks to many, many, many months waiting on nursing. Which is certainly not good for the child. Not good for the family, not good for the hospital that has a shortage of beds. And not good for the state, assuming they’re actually paying like an inpatient floor PICU stay, you know, during that time.” – *Provider_1* *“*We try to maintain as normal a life as possible…If you look back 30 years ago, my daughter would not be in our home. My daughter would be in a facility, and the state would be footing the bill…She is a level three medically-fragile child, and just because we don’t have nursing, our life doesn’t stop. We continue, we adapt, we overcome.” – *Caregiver_1*
*Reduce family financial burden*	“You can just imagine a situation where a baby has devastating problems in the NICU. A young couple looks at each other and has to decide who’s not going back to work…If you’re not poor before, you can become poor…And part of that is you do wind up paying for things out of pocket, in terms of medical care expenses, etc., but you also lose income.” – *Provider_3* “I’m able to go to work. I would not be able to work if I didn’t have the nurse. I would have to give up my work.” – *Caregiver_7*

In addition, caregivers described their efforts to maintain “normal lives” for their families and the full-time care needs of CMC that were previously addressed and financed by the state. When asked what they wish healthcare providers understood about their role as a caregiver of CMC, caregivers frequently reported wishing providers were more understanding of the difficulty and instability of their role and its impact on their daily lives—despite their unwavering love for their children.

Reducing family financial burden would significantly improve the QoL of CMC and their families (Table 4). Physicians and caregivers cited accessible home nursing care as an opportunity for caregivers to maintain employment. Employment opportunities for caregivers of CMC may allow these families to reduce the financial stress associated with CMC’s medical needs and remain engaged in a purpose outside of the home. Otherwise, in many cases among CMC without accessible home nursing care, physicians explained the loss of significant household income and subsequent financial instability to care for CMC full-time. In agreeance, caregivers described their reliance on home nursing care to maintain employment. Caregivers with flexible employers reported being better able to maintain their home nursing care, as home nursing care inevitably fluctuates, and caregiving responsibilities take priority over responsibilities outside the home. These caregivers may also have more flexibility to attend counseling, support groups, and other community-based support resources currently reported as inaccessible due to the financial cost and lack of available respite care.

## Discussion

This study’s findings suggest that patient-level indicators predict hospital admission(s), and hospitalizations demonstrate dynamic, bidirectional relationships with economic factors, patient and family well-being and QoL, and overlapping concepts of quality of home-based medical care (Fig. 1). Home-based medical care, provided by family members or home nurses, involves overlapping concepts of access (or lack of) home nursing care, complexity of care, capacity, and willingness of caregivers. These concepts hold complex, bidirectional relationships with financial factors and patient and family well-being and QoL. In addition, economic factors directly impact patient and family well-being and QoL.

### Providing complex care in the home environment

In light of the effects of COVID-19, there is a national emphasis on the need for social and emotional support to address increased caregiver distress [31–33]. Caregivers can often provide the highest quality healthcare, yet the reality of current support among CMC and their families is alarming [18, 19, 34, 35]. Our findings suggest that CMC who are reliant on respiratory and/or neurological/neuromuscular medical devices, who have visited the ED in the past 12 months, and who are younger than 3 years old are approximately 18 to 28% more likely to experience at least one HA, compared to CMC who are reliant on renal or urologic devices, without an ED visit, or who are at least 3 years old. While this may be partially explained by the severity of the child’s condition, CMC and their families in these groups may benefit from tailored support and specialized training in the home to prevent hospitalizations.

Caregivers in this study convey the need for more social-emotional support, while acknowledging the complex level of care required by their children. Previous research found CMC had lower satisfaction with healthcare services than CYSHCN with less complex conditions and most frequently sought outpatient services for mental healthcare, which may also reflect the mental health of caregivers in the household [2, 36]. Physicians reiterate concerns for CMC and caregiver mental health and well-being, and express feelings of helplessness in their abilities to connect CMC and their families with the resources they need due to staffing shortages, complexity of patient conditions, SDOH, and bureaucracies of the referral process. While caregivers of CMC are more likely to report receiving emotional support from healthcare physicians and advocacy groups, compared to caregivers of non-complex CYSHCN; caregivers in this study identify financial and time constraints as barriers to participating in such resources [36]. Family support between the health system and community-based resources is needed to partner with caregivers of CMC and identify and implement accessible opportunities for social-emotional support across SC. Virtual options, including social media platforms, have been suggested by caregivers in this study as an opportunity to connect statewide without the transportation, time, and financial barriers of traveling to an in-person support group [37].

### SDOH considerations

The influence of SDOH factors on CMC and their families is well-documented, particularly regarding household financial burden and caregiver employment and well-being [5, 38, 39]. This study found no significant associations between patient’s race/ethnicity and hospitalization(s). This is contrary to the literature, which suggests children with the highest complexity of care and who identify with historically marginalized communities may be at a heightened risk of experiencing fragmented healthcare services and ongoing unmet healthcare needs—creating a ripple effect that affects CMC’s caregivers and other family members [3, 40].

Surprisingly, this study found no association between SDOH position and HA risk. However, this may be a limitation of the SDOH measure existing at the neighborhood-level instead of individual-level, rather than the lack of relationship between these factors, as has been documented in similar literature [41]. Physicians and caregivers in this study reiterate the significance of job security and flexibility, poverty and financial considerations, time constraints, and mental health of caregivers when illustrating the experiences of this sample population. Still, this analysis identifies CMC who visit the ED and are reliant on neurological/neuromuscular or respiratory medical devices as more likely to be hospitalized, which are documented risk factors for financial burden in existing research [22, 38]. Several care coordination models have been proposed to address health inequities and SDOH barriers among CMC and their families; however, implementation science and comparative effectiveness research is needed [3, 12, 17, 35, 38]. In recent years, a research agenda for implementation and dissemination of care models among this population supports the system-level transition from “spending to investment” as a national priority [17]. Sustainable, investment-oriented care models include the coordination of efficient healthcare services that meet children’s needs and prioritize their long-term health outcomes—sometimes at the cost of short-term expenses [17].

Further, nearly half (46.77%) of this sample population live in a zip code with a “very low,” “low,” or “moderate” opportunity level, which aligns with previous studies that CMC are disproportionately at-risk of living in poverty [22, 42]. Future research is needed for household-level measures of SDOH impact and should be used to consider interventions, such as tailored care coordination models, to improve healthcare access with minimal burden on caregivers of CMC [35, 43–45]. Additional opportunities for caregiver respite, despite lacking home nursing care and SDOH, should be investigated to support this population.

### Systemic and policy-level barriers to home nursing care

In recent years, the national healthcare system has demonstrated pioneering resiliency to meet patient’s needs among vulnerable populations, such as CMC [28]. In alignment with agendas for pediatric healthcare reform, CMC and their families have been invited to identify and solve historical, ongoing barriers in the health system to improve well-being and QoL among this population [27]. According to this study’s findings, accessible home nursing care may dramatically affect the daily lives of CMC and their families in SC. However, as is partially explained by the shifting landscape of the nursing profession since the onset of COVID-19, barriers to home nursing care—including staffing shortages, financial factors, and lack of capacity of nurses—among CMC in SC persist.

Physicians and caregivers of CMC in this study unanimously identified compensation among home health nurses as a significant contributing factor to the nursing shortage, which is consistent with national home nursing studies [6, 11]. In less urban areas, previous research suggests reimbursement for home nursing care may be as low as $19 per hour, leaving home nurses susceptible to compassion fatigue, burnout, and personal financial distress [6]. Further, physicians in this study describe the expensive and unnecessarily long PICU stays that CMC and their families often experience in SC due to the lack of support in the home environment. Another multi-site study among 1,582 CMC spent $5.72 million in hospital costs due to prolonged delays over 12 months, compared to the estimated $769,326 if home healthcare had been available [6, 46]. In alignment with the ongoing shift from a spending to an investment-oriented approach, health systems should consider the implementation of several evidence-based, systemic interventions, including expansion of telehealth, integration between inpatient and home healthcare settings, student loan repayment, payment reform for improved compensation and student and post-graduate training for home health to prevent hospitalizations and equitable community-based supports for caregivers of CMC [17, 19, 36, 46–48]. While family advocates continue to push for these interventions and policy changes, more legislative awareness is needed to make substantial, sustainable progress toward a supported healthcare system for CMC and their families [1, 3, 27, 35].

Further, this study’s findings contextualize the reliance on caregivers to fill clinical care gaps statewide and emphasize caregivers’ capacity and willingness to care for CMC. Caregivers repeatedly report that accessible nurses may not be appropriately trained or willing to provide highly complex care (referring to their “capacity” to meet patient needs), and as a result, caregivers often cannot work due to their children’s care needs. Previous research has studied the opportunity to financially reimburse family caregivers of CMC due to foregone employment in an attempt to reduce financial and SDOH barriers among this population [6, 12]. Family training at the bedside during hospitalizations has also effectively reduced caregiver distress and improved home-based support in previous research [19]. Further, existing research cites the variability in agencies’ hiring and training practices as a limitation for home health nurses, contributing to issues with retention and long-term support of families of CMC [49]. Paired with training and certification opportunities for caregivers and home nurses in patient-specific care needs, this proposed intervention may mediate the financial and household-level burden on caregivers of CMC for those with and without accessible home nursing care alike.

Regardless of the financial compensation for caregivers of CMC, this study’s findings echo the need to acknowledge caregivers’ daily responsibilities to improve emotional, social, and financial well-being and to fill the gaps in home nursing care [18, 35, 50, 51]. Peer support and parenting programs, targeted behavior change, and mental health intervention among caregivers have been suggested to improve caregiver health in previous studies [19, 50]. Further caregiver research is needed to identify and implement social-emotional supports across SC, with needs and preferences of this population as key to driving the implementation of statewide supports.

### Limitations

Due to the sensitive nature of the study and the prevalence of CMC in the general population, this sample size is small and limited to one health system [1, 3]. This may limit the generalizability of quantitative findings, such as the consolidation of race and/or ethnicity into a binary variable and the low variability of zip code-level estimates. Additionally, within the electronic health record, the primary care office where this data was extracted does not record those on the Medicaid Medical Complexity Waiver [52], or who are referred to and/or receive home nursing care. Therefore, this analysis was limited in its ability to identify risk factors for referral and access to home nursing care in this region.

### Future directions for CMC research

This study’s approach prioritized the inclusion of caregiver and provider perspectives to initiate exploration into factors associated with HA and unmet healthcare needs. Future work among this research team intends to broaden its reach by requesting statewide claims data and developing relationships with key stakeholders statewide. To improve generalizability and comparative analyses, similar research is suggested within additional geographical regions or across multiple states/healthcare regions.

## Conclusions

Integration of quantitative findings with caregivers and providers of CMC perspectives provides insight into the unmet needs of this population and depth to challenges that may be masked by the nature of pediatric complex care. Although findings in this study and similar studies found associations between HA risk and patient-level factors, including care needs and race/ethnicity, the inclusion of caregiver and provider testimony highlights the impact of HA and various mechanisms needed to reduce this risk. Similarly, while SDOH was not found to have an association with HA at the neighborhood level, the prevalence of CMC living in “low” or “very low” opportunity, coupled with caregivers’ narrative regarding the burden of home care and balancing life with CMC, should be used to explore the impact of caring for CMC.

Improving healthcare access, including home nursing care, for CMC and their families requires work at several levels. Implications of this study and similar research have begun to address barriers at different levels in the care pathway. At the practice level, providers continued support for caregivers and connection to services is vital. At the policy level, advocacy for multi-level interventions is needed to address the systemic barriers to care among children and families who are most vulnerable. Additionally, advocating for healthcare systems that can support skilled, high-quality home nurses with adequate compensation and payer models that support family choice. These findings should be used in future research to systematically uncover opportunities to address barriers and enhance factors of healthcare systems that support optimal outcomes for CMC and their families, as well as their healthcare team.

### Electronic supplementary material

Below is the link to the electronic supplementary material.

Supplementary Material 1

## Data Availability

Patient-level data is not available due to confidentiality and privacy laws. De-identified quantitative and qualitative data is provided in the Tables throughout this manuscript. Interview guides used for data collection are included as appendices.
