# Supplementary material for: Bridging the gaps in pediatric complex healthcare: the case for home nursing care among children with medical complexity
Source: BMC Health Serv Res. 2024 Jul 15;24:814. doi: 10.1186/s12913-024-11235-1 (PMC11251302; doi:10.1186/s12913-024-11235-1)
Supplement: Supplementary file 1 — Supplementary Material 1 [file 12913_2024_11235_MOESM1_ESM.docx]

| **Appendix A: Categorization of Medical Devices by Body System (using ICD-10 Codes)** | |
| --- | --- |
| Neurologic & Neuromuscular Devices | T85.09XA, T85.190A, T85.192A, T85.199A, T85.79XA, Z98.2, Z45.41, Z45.42 |
| Cardiovascular Devices | T82.519A, T82.529A, T82.539A, T82.599A, T82.110A, T82.111A, T82.120A, T82.121A, T82.190A, T82.191A, T82.01XA, T82.02XA, T82.03XA, T82.09XA, T82.211A, T82.212A, T82.213A, T82.218A, T82.221A, T82.222A, T82.223A, T82.228A, T82.518A, T82.528A, T82.538A, T82.598A, T82.6XXA, T82.7XXA, Z95.0, Z95.2, Z95.3, Z95.810-Z95.812, Z95.818, Z45.010, Z45.018, Z45.02, Z45.09, Z95.9 |
| Respiratory Devices | J95.00-J95.04, J95.09, Z43.0, Z93.0, Z99.0, J95.850, Z99.11, Z99.12 |
| Renal & Urological Devices | T85.71XA, Z93.50-Z93.52, Z93.59, Z93.6, Z91.15, Z99.2, Z43.5, Z43.6, Z46.6 |
| Gastrointestinal Devices | K94.20, K94.22, K94.23, K94.29, Z93.1-Z93.4, Z43.1-Z43.4, Z46.51, Z46.59 |

| **Appendix B: Childhood Opportunity Index 2.0 Indicators (Domain: Social and Economic).** | | |
| --- | --- | --- |
| **Subdomain** | **Indicator** | **Operationalization** |
| Economic opportunities | Employment rate | Percentage of adults aged 25-54 who are employed |
|  | Commute duration | Percentage of workers commuting more than one hour one way* |
| Economic and social resources | Poverty rate | Percentage of individuals living in households with incomes below 100% of the federal poverty threshold* |
|  | Public assistance rate | Percentage of households receiving cash public assistance or Food Stamps/Supplemental Nutrition Assistance Program* |
|  | Homeownership rate | Percentage of owner-occupied housing units |
|  | High-skill employment | Percentage of individuals aged 16 or older who are employed in management, business, financial, computer, engineering, science, education, legal, community service, healthcare practitioner, health technology, arts and media occupations |
|  | Median household income | Median income of all households |
|  | Single-headed households | Percentage of family households that are single-parent headed* |

*Identifies indicators that are reverse coded, so higher values represent higher opportunity

**Appendix C: Interview Guides for Physicians and Family Caregivers of Children with Medical Complexity**

*For physicians*

**Introduction**

- Tell me about your experience with home-based skilled nursing services. (Probes: accessibility, referral process, etc.)
- What qualifies your patients to be referred for home-based skilled nursing visits?
- How often do your patients have difficulty accessing referred home-based nursing visits?*,***
  - Do certain groups have more trouble receiving referred home-based nursing than others? Tell me more about that. (Probes: location/rurality, racial/ethnic differences, etc.)
- In your opinion, what could be done to improve access to home-based skilled nursing services for CMC and their families? (Probe: policy/etc.; ex. staffing, reimbursement, care coordination services)***,****

**Perceived value**

- In your opinion, what are the benefits of home health nursing for CMC and their families? (Probes: child, caregiver, siblings, household/family function)**
- How might home-based skilled nursing influence the health and well-being of CMC? Tell me more about that.**,***
  - How might it influence the health and well-being of caregivers? Siblings/other family members?
  - How might accessible home-based skilled nursing influence the long-term health outcomes of CMC?*,**,***
  - How might it influence the long-term health outcomes of their caregivers? Siblings? (Probes: household/family function)**

How do you perceive the value of home-based skilled nursing vs. home health aide services?

- Are there differences in accessibility between these services?***

**Facilitators/Barriers**

- What factors do you believe influence a child/family’s likelihood of receiving home-based skilled nursing services when they are needed?*,**,***,****
- What is your role in helping connect CMC with home-based skilled nursing services?***
  - What factors are helpful when connecting with home nursing?
  - Do certain groups/areas experience these factors more than others? Tell me more about that.
- What are the biggest barriers, in your opinion, that CMC and their families experience when seeking home nursing services?*,**,***,****
  - What are the barriers of this care being effective? (Probe: sustaining access, ongoing care, home set up; Note: combination of system/family/pt side)
  - Do certain groups/areas experience these factors more than others? Tell me more about that.
  - In your opinion, what could be done to address these barriers and make services more accessible? (Probe: local, regional, state/nationwide)

**Conclusion**

- How are you able to use your clinical position to advocate for your patients and their families in these situations?*,***
- How would you feel better supported as a clinician to provide optimal quality of care to your patients and their families?**,***

*Notes:*

*Denotes questions related to “Critical Area 1: Health equity”

** Denotes questions related to “Critical Area 2: Family and child well-being and quality of life”

***Denotes questions related to “Critical Area 3: Access to services”

**** Denotes questions related to “Critical Area 4: Financing of services”

*For caregivers*

**Introduction**

- Describe your role as a caregiver for your child. (Probes: Medical complexity/reliance on technology, daily routines, etc.)**
- How many hours (per day/week) do you provide hands-on medical care for your child?
  - How often do you feel like a trusted partner as part of your child’s healthcare team?**
  - Conversely, how often do you feel like you are not a trusted partner in your child’s healthcare team?
- How well do you believe your child’s provider understands the role that your caregiving responsibilities impact your daily life?**
- Conversely, tell me about a time when you felt your child’s provider did not understand the role that your caregiving responsibilities impact your life.
  - If you have concerns about your child’s medical care or your ability to manage your caregiving responsibilities at home, who can you talk to?
  - Probe: Can you tell me about the areas where your child’s provider doesn’t understand your role?
  - How often do providers check in on you and your health/capacity as a caregiver?
- What could be done so that you felt better supported in your daily life?

**Value/Benefits**

- Has your child ever been referred to receive home nursing services?
  - What was the reason for the referral?
  - Was your child able to receive the referred services?***
  - Can you tell me more about that process went?
- In your opinion, how did these (/would these) home nursing services impact your child’s health condition?**,****
  - How did these (/would these) home nursing services impact your caregiving responsibilities?
  - How might home nursing services help carryover your child’s care between the healthcare system and your home environment?
- In your opinion, what role might home nursing services play in your child and family’s well-being (and/or quality of life)?**,****
- How do you (and your child) feel supported by your child’s healthcare team in your daily life?

**Facilitators/Barriers**

- What factors do you believe helped (or would have helped) you and your family access home-based nursing services when they are needed? Tell me more about that. *,**,***,****
- How did your child’s healthcare team support you in connecting to home-based skilled nursing services?
  - What (if anything) would you change about the way your child’s healthcare team supported you through this process?**
  - Who do you feel that you can talk to if you are struggling with accessing these services?**
- What are the biggest barriers that you/your family experienced when seeking home nursing services?*,**,***,****
  - In your opinion, what could be done to address these barriers and make services more accessible? (Probe: local, regional, state/nationwide)

**Conclusion**

- What do you wish providers/clinicians understood about your role as a caregiver of a child with medical complexity?

Notes:

*Denotes questions related to “Critical Area 1: Health equity”

** Denotes questions related to “Critical Area 2: Family and child well-being and quality of life”

***Denotes questions related to “Critical Area 3: Access to services”

**** Denotes questions related to “Critical Area 4: Financing of services”
